# In Hospital Assessment and Management of High Bleeding Risk in Patients with ST-Elevation Myocardial Infarction (STEMI)

**DOI:** 10.3390/jcdd13050183

**Published:** 2026-04-27

**Authors:** Sanghoon Kim, Alberto Piserra-López, Salvatore Giordano, Claudio Laudani, Francesco Costa, Nelsa González-Aguado, Nicola Corcione, Dominick J. Angiolillo, Luis Ortega-Paz

**Affiliations:** 1 Department of Cardiology, CHA Bundang Medical Center, CHA University, Seongnam 13497, Republic of Korea; kimsang97@cha.ac.kr; 2 Division of Cardiology, College of Medicine-Jacksonville, University of Florida, Jacksonville, FL 32209, USA; 3 Área del Corazón, Hospital Universitario Virgen de la Victoria, IBIMA Plataforma BIONAND, 29010 Malaga, Spain; alberto.piserra.lopez@gmail.com (A.P.-L.); nelsa.gonzalez.sspa@juntadeandalucia.es (N.G.-A.); 4 Centro de Investigación Biomédica en Red de Enfermedades Cardiovasculares (CIBERCV), 28029 Madrid, Spain; 5 Department of Medical and Surgical Sciences, Division of Cardiology, “Magna Graecia” University, 88100 Catanzaro, Italy; 6 Division of Cardiology, Azienda Ospedaliero-Universitaria Policlinico “Rodolico-San Marco”, University of Catania, 95123 Catania, Italy; 7 Invasive Cardiology Unit, Pineta Grande Hospital, Castel Volturno, 81030 Caserta, Italy

**Keywords:** ST-elevation myocardial infarction (STEMI), high bleeding risk, bleeding risk stratification, antithrombotic therapy, percutaneous coronary intervention

## Abstract

Bleeding risk assessment is a critical component of the management of patients with ST-segment elevation myocardial infarction (STEMI), yet the optimal approach to risk stratification remains controversial. Although several bleeding risk scores have been developed, their predictive performance in STEMI populations is still evolving. Importantly, bleeding risk in STEMI is dynamic and influenced by clinical status, procedural factors, and antithrombotic strategies, underscoring the need for continuous reassessment throughout hospitalization. Bleeding avoidance measures—including radial access, judicious use of anticoagulation, and individualized antiplatelet therapy—play a pivotal role in reducing complications. Balancing ischemic and hemorrhagic risks is particularly challenging in patients with concomitantly high thrombotic and bleeding risks, requiring tailored management strategies. As bleeding remains a major determinant of prognosis, refining risk stratification tools and integrating evidence-based bleeding prevention strategies into clinical practice are essential. This narrative review summarizes the current evidence regarding the identification of high bleeding risk in hospitalized patients with STEMI and discusses its clinical implications. Also, this review proposes a dynamic, phase-specific framework for in-hospital bleeding risk assessment and management in patients with STEMI.

## 1. Introduction

ST-elevation myocardial infarction (STEMI) remains a critical cardiovascular emergency. Over recent decades, mortality and morbidity have been significantly reduced through timely reperfusion strategies (primary percutaneous coronary intervention or fibrinolysis), optimized antithrombotic therapy, and advances in supportive care [1,2]. However, these therapeutic improvements have been accompanied by an increased risk of bleeding, particularly in patients with multiple comorbidities, making individualized assessment and management essential in those at high bleeding risk.

Major bleeding in the setting of STEMI is associated with increased in-hospital mortality, reinfarction, stroke, prolonged hospitalization, need for transfusion, and worse long-term outcomes [3]. Importantly, bleeding events occurring early after presentation or intervention appear to carry a particularly adverse prognostic impact, underscoring bleeding prevention as a cornerstone of early STEMI management [4].

In clinical practice, high bleeding risk (HBR) frequently coexists with high ischemic risk, creating a complex therapeutic trade-off. Although several bleeding risk scores and trade-off models have been developed over the last decade to support personalized decision-making, their applicability and performance in patients with STEMI remain limited. Consequently, accurate identification of HBR and tailored management strategies are crucial to optimize outcomes in this high-risk population.

This narrative review was performed to summarize the current evidence on the identification of HBR in hospitalized patients with STEMI. A literature search was conducted using PubMed, Embase, and Google Scholar using keywords including “STEMI”, “high bleeding risk” and “bleeding risk stratification”. Relevant articles were selected based on their relevance to the subject and were reviewed to provide a comprehensive overview of the topic. Also discusses practical strategies to optimize in-hospital management, including pharmacological choices, procedural considerations, monitoring, and unmet knowledge gaps. A dynamic, phase-specific approach to bleeding risk assessment is emphasized.

## 2. Defining High Bleeding Risk: What, Why and How

### 2.1. Why It Matters in STEMI

- A substantial proportion of patients with STEMI meet criteria for high bleeding risk at presentation.
- Management of STEMI requires exposure to multiple antithrombotic agents, resulting in a high cumulative bleeding burden.
- Hemodynamic instability and frequent comorbidities increase susceptibility to bleeding complications.
- Bleeding events are independently associated with adverse clinical outcomes, including increased mortality and morbidity.

Bleeding represents a clinically relevant complication in patients with acute coronary syndromes, and accumulating evidence indicates that in-hospital bleeding is not only frequent but also an independent predictor of both short- and long-term mortality among patients with ST-elevation myocardial infarction (STEMI) [5,6]. While advances in reperfusion strategies and the widespread use of potent antithrombotic agents have substantially improved ischemic outcomes, these same therapies have inevitably increased the incidence of bleeding complications [7,8].

Importantly, both early hemorrhagic events occurring during the acute or periprocedural phase and late bleeding developing during long-term antithrombotic therapy carry significant prognostic implications. This underscores the need to consider bleeding risk across the entire spectrum of acute coronary syndrome (ACS) management, rather than as a secondary safety concern.

High bleeding risk (HBR) is highly prevalent among patients presenting with STEMI. In a contemporary real-world cohort, 42.7% of STEMI patients fulfilled the Academic Research Consortium for High Bleeding Risk (ARC-HBR) criteria. The most frequent minor criteria included advanced age (≥75 years), moderate chronic kidney disease, and mild anemia, whereas the most common major criteria were oral anticoagulation, moderate-to-severe anemia, advanced chronic kidney disease, bleeding diathesis, and active malignancy. These findings highlight the substantial epidemiological burden and heterogeneity of bleeding risk factors in this population, posing significant challenges when balancing ischemic protection against bleeding prevention [9].

A large contemporary systematic review and meta-analysis including more than 140,000 patients with coronary artery disease demonstrated that major bleeding is associated with an excess risk of all-cause mortality comparable to that of myocardial infarction. When stratified by timing, early bleeding—defined as periprocedural or occurring within the first 30 days after ACS or revascularization—was associated with a significantly higher mortality risk than early myocardial infarction, whereas late bleeding and late myocardial infarction conveyed a similar prognostic impact. In contrast, minor bleeding was associated with substantially lower mortality. These data establish bleeding, particularly when occurring early, as a major prognostic determinant in the acute phase of ACS rather than a mere safety endpoint [4].

Consistent with these observations, the randomized TRACER trial showed that both myocardial infarction and clinically relevant bleeding events (BARC ≥ 2) occurring beyond 30 days after ACS were independently associated with increased mortality, with a graded relationship according to bleeding severity. While myocardial infarction conferred a higher mortality risk than moderate bleeding, major bleeding (BARC ≥ 3)—especially intracranial hemorrhage—had a prognostic impact comparable to or exceeding that of myocardial infarction, with both event types showing a pronounced early hazard after occurrence [10].

More recent observational analysis derived from the PRAISE registry including 23,270 patients hospitalized for ACS, 1060 patients experienced in hospital bleeding (IHB) during the index hospitalization. Patients with IHB were generally older, more often female, and had a higher burden of comorbidities compared with those without bleeding. Importantly, the occurrence of IHB was associated with a lower likelihood of receiving optimal evidence-based therapies at discharge and with significantly worse clinical outcomes at one year, including higher rates of all-cause mortality and major bleeding. Although the association with reinfarction was attenuated after multivariable adjustment, IHB remained a strong marker of adverse prognosis. These findings underscore the importance of preventing bleeding during hospitalization, particularly in high-risk populations such as patients with STEMI who require intensive antithrombotic therapy [11].

In routine clinical practice, bleeding events frequently lead to premature discontinuation of dual antiplatelet therapy, delays in invasive procedures, and prolonged hospitalization, all of which may adversely affect outcomes [12]. Consequently, systematic identification and individualized management of bleeding risk have become integral components of contemporary STEMI care, aiming to achieve an optimal balance between efficacy and safety [13].

### 2.2. Current Guideline Recommendations for Bleeding Risk Stratification in STEMI

#### 2.2.1. 2023 ESC Guidelines for ACS

The 2023 European Society of Cardiology (ESC) guidelines endorse a structured assessment of bleeding risk to guide the intensity and duration of antithrombotic therapy in patients with acute coronary syndromes. In particular, contemporary consensus definitions of high bleeding risk—most notably the Academic Research Consortium for High Bleeding Risk (ARC-HBR) criteria—are recognized as useful tools for clinical decision-making, as they identify patients with an estimated ≥4% 1-year risk of major bleeding (BARC 3–5) or ≥1% risk of intracranial hemorrhage. In addition, validated bleeding risk scores, such as PRECISE-DAPT and related constructs, are recommended as adjuncts to clinical judgment when individualizing dual antiplatelet therapy (DAPT) duration after percutaneous coronary intervention (PCI) [13].

For patients with STEMI classified as having high bleeding risk, the ESC recommends several in-hospital strategies aimed at reducing bleeding without compromising reperfusion efficacy. These include preferential use of transradial arterial access when feasible to minimize access-site bleeding, avoidance of routine pretreatment with P2Y_12_ receptor inhibitors, restriction of glycoprotein IIb/IIIa inhibitors to bailout situations or the management of periprocedural complications, careful consideration of bleeding risk when selecting the type and duration of antiplatelet therapy, and routine co-prescription of gastroprotective therapy with proton pump inhibitors when clinically appropriate.

Regarding antiplatelet therapy, the guidelines propose specific DAPT strategies for patients at high bleeding risk, including abbreviated and de-escalation approaches. Among abbreviated strategies, a 3- to 6-month DAPT duration in patients with high bleeding risk and no high ischemic risk is recommended with a class IIa, level of evidence A. An even shorter 1-month DAPT regimen may be considered in selected patients (class IIb, level of evidence B). De-escalation from a potent P2Y_12_ inhibitor (ticagrelor or prasugrel) to clopidogrel after one month to reduce bleeding risk carries a class IIb recommendation with level of evidence A, whereas routine de-escalation within the first 30 days is not currently recommended.

The ESC guidelines also address the management of patients requiring combined antithrombotic indications, such as those with atrial fibrillation on chronic oral anticoagulation. In this context, PCI may be performed without interruption of vitamin K antagonists or non–vitamin K oral anticoagulants, with tailored use of additional parenteral anticoagulation. The duration of triple antithrombotic therapy should be minimized, favoring early discontinuation of aspirin—often after one week—followed by 6 to 12 months of treatment with oral anticoagulation plus a P2Y_12_ inhibitor, individualized according to the balance between bleeding and thrombotic risk.

Finally, the ESC emphasizes that discharge planning in STEMI patients at high bleeding risk should include explicit documentation of bleeding-risk considerations, appropriate follow-up arrangements, and patient education regarding signs and symptoms of bleeding [14].

#### 2.2.2. 2025 ACC/AHA/ACEP/NAEMSP/SCAI Guideline for ACS

The 2025 ACC/AHA guideline emphasizes early identification and individualized management of high bleeding risk in patients with STEMI, integrating ischemic and hemorrhagic risk to guide antithrombotic therapy. Radial arterial access is recommended as the default strategy during primary PCI to minimize access-site bleeding. When femoral access is required, ultrasound-guided arterial puncture is advised. In addition, the use of glycoprotein IIb/IIIa inhibitors should be restricted to bailout situations, such as high thrombotic burden or no-reflow phenomena. Careful selection and dose adjustment of parenteral anticoagulants are recommended, particularly in elderly patients and those with renal dysfunction.

With respect to antiplatelet therapy, a tailored approach is advocated, favoring shorter durations of DAPT and early de-escalation in patients at high bleeding risk, while preserving ischemic protection. Several bleeding-reduction strategies are endorsed. With a class I recommendation, ticagrelor monotherapy after one month of DAPT with ticagrelor and aspirin is recommended. In patients requiring long-term oral anticoagulation, triple therapy with oral anticoagulation, aspirin, and clopidogrel should be limited to one week, followed by dual therapy with oral anticoagulation and clopidogrel for up to one year.

With a lower level of recommendation (class IIb), de-escalation strategies may be considered, including switching from potent P2Y_12_ inhibitors plus aspirin to clopidogrel plus aspirin after one month. In selected patients at high ischemic risk, discontinuation of DAPT from one month onwards with continuation of single antiplatelet therapy—either aspirin or a P2Y_12_ inhibitor—may also be considered.

Both European and American guidelines concur that while prasugrel or ticagrelor should generally be preferred as P2Y_12_ inhibitors in STEMI, clopidogrel remains the recommended second antiplatelet agent when fibrinolysis is used as the initial reperfusion strategy. Routine use of bleeding risk scores and early gastroprotective measures, including proton pump inhibitors, is encouraged. Finally, the guideline underscores the importance of multidisciplinary decision-making and structured in-hospital monitoring to balance reperfusion efficacy with bleeding avoidance, aiming to improve both short- and long-term outcomes in this high-risk population [1].

#### 2.2.3. Bleeding Risk Scores in STEMI

Most currently available bleeding risk scores were developed to estimate post-discharge bleeding risk and guide antiplatelet therapy management. Importantly, the majority of these tools were derived from cohorts of patients with stable coronary artery disease or mixed acute coronary syndrome populations, in which patients with STEMI were underrepresented. Moreover, validation cohorts largely included stable patients receiving dual antiplatelet therapy or patients with non–ST-elevation myocardial infarction, limiting the extrapolation of their predictive performance to the STEMI setting [15,16].

Although bleeding risk scores such as CRUSADE, ACTION, ACUITY-HORIZONS, and SWEDEHEART are not routinely applied in daily clinical practice, data on their predictive performance in STEMI patients—particularly from Asian cohorts—are available (Table 1). In a study of Chinese patients undergoing primary PCI, both the CRUSADE and ACTION scores demonstrated good discrimination for in-hospital major bleeding and showed a stepwise increase in mortality across bleeding risk categories at one year. In contrast, the ACUITY-HORIZONS score exhibited weaker discrimination and suboptimal calibration, supporting the relative robustness of CRUSADE and ACTION in this clinical context [17].

The ACTION Registry–GWTG bleeding risk score relies exclusively on baseline clinical variables available at presentation, making it practical in emergency settings. Derived from a large U.S. registry, it demonstrated good predictive accuracy, with consistent performance in STEMI patients (AUC ≈ 0.70). Bleeding rates ranged from 3.9% in the lowest-risk quintile to nearly 40% in the highest-risk group [18].

More recently, the SWEDEHEART bleeding risk score was developed using data from a large nationwide registry. The simplified model incorporates a limited number of readily available variables and demonstrated high predictive accuracy (C-index ≈ 0.80), outperforming recalibrated CRUSADE and ACTION scores. Its derivation from a contemporary real-world population enhances generalizability, and the inclusion of inflammatory markers represents an incremental improvement over prior models [19].

Collectively, these findings indicate that while CRUSADE and ACTION remain useful tools for bleeding risk stratification in STEMI, SWEDEHEART represents a contemporary advancement with improved predictive performance. Nonetheless, the overall discriminative ability of currently available scores remains moderate, underscoring the need for bleeding risk models specifically tailored to the STEMI population.

## 3. Currently Employed Bleeding Risk Scores in STEMI Patients

### 3.1. PRECISE-DAPT

Among currently employed bleeding risk scores the PRECISE- DAPT score has proven to be predictive of in-hospital mortality in patients with STEMI, as well as for major bleeding events. The PRECISE-DAPT score was published in 2017 as a tool to gauge out-of-hospital bleeding risk in patients on DAPT who underwent PCI. It is a collaborative study including a total of 14,963 patients, pooled from 8 RCTs conducted between 2006 and 2014. The cohort included a heterogeneous population (including patients from Europe, America, and East Asia) who underwent elective, urgent, or emergent PCI with coronary stent implantation and who were treated with DAPT (mostly clopidogrel, 88%) [12].

The predictors individuated were five: age, creatinine clearance, hemoglobin, white-blood-cell count at baseline, and previous spontaneous bleeding. Points were assigned to each of these variables based on the magnitude of association of each predictor with bleeding and were integrated into a score (available at www.precisedaptscore.com). A score higher or equal to 25 indicates patients at increased risk of bleeding [20].

In a cohort of 1418 patients with STEMI undergoing primary PCI, those with a PRECISE-DAPT score ≥ 25 had significantly higher in-hospital mortality compared to those with a score < 25 (9.4% vs. 0.9%, *p* < 0.001). The score also correlated with an increased incidence of major bleeding (5.6% vs. 0.9%, *p* < 0.001). On multivariate analysis, the PRECISE-DAPT score remained an independent predictor of in-hospital mortality (HR: 1.026, 95% CI: 1.004–1.048; *p* = 0.021). Given that all required parameters are readily available on admission, the PRECISE-DAPT score offers a simple yet powerful tool not only for estimating bleeding risk but also for early mortality risk stratification in STEMI patients undergoing primary PCI [21].

Cancer is a recognized factor associated with an increased bleeding risk in patients with STEMI [22]. In this context, the recently introduced PRECISE-DAPT Cancer Score incorporates cancer as an additional binary variable into the original PRECISE-DAPT model, leading to improved bleeding risk stratification in STEMI patients with cancer [23]. This modified score reclassifies a substantially higher proportion of these patients as high bleeding risk (94% vs. 65.5% with the original score) and enhances overall discriminatory performance, while maintaining accuracy in patients without cancer. This novel score has been recently externally validated in multiple cohorts showing decent discrimination in patients with and without cancer [24,25].

### 3.2. ARC-HBR (Academic Research Consortium for High Bleeding Risk)

The ARC-HBR framework is widely adopted and supported by both European and American clinical practice guidelines as a standardized approach for identifying patients at increased bleeding risk in research and routine clinical practice. It is based on the presence of major and minor clinical criteria, and patients are classified as having high bleeding risk when at least one major or two minor criteria are fulfilled. This framework defines high bleeding risk as an estimated ≥4% incidence of BARC 3 or 5 bleeding and/or ≥1% risk of intracranial hemorrhage at one year [26]. These thresholds were derived from observations in DAPT trials, in which patients without bleeding-related features exhibited one-year major bleeding rates below 3%, whereas those with such features showed rates ranging from approximately 3.5% to 7.2% [27,28,29,30,31,32].

More recently, a hybrid bleeding risk model (PRECISE-HBR) incorporating ARC-HBR criteria into the original PRECISE-DAPT framework has been developed. This score integrates seven variables and identifies patients at high bleeding risk using a cut-off value of 23, corresponding to a ≥4% incidence of BARC 3 or 5 bleeding at one year. In validation cohorts, PRECISE-HBR demonstrated superior discriminatory performance compared with PRECISE-DAPT and other established bleeding risk scores [33].

Despite its broad adoption, the real-world performance of ARC-HBR in patients with STEMI remains an area of active investigation. Several studies have specifically evaluated its predictive ability in this high-risk population, assessing both in-hospital and longer-term bleeding outcomes.

In a Japanese single-center study including 939 STEMI patients undergoing PCI, 42.9% fulfilled ARC-HBR criteria. Major bleeding occurred significantly more frequently in HBR patients than in non-HBR patients (13.7% vs. 6.7%, *p* < 0.01). Overall discrimination of ARC-HBR for predicting bleeding events was modest (C-statistic 0.60) and inferior to that of PRECISE-DAPT (0.69, *p* < 0.01). Notably, after exclusion of patients receiving mechanical circulatory support—who exhibited high bleeding rates irrespective of baseline risk—the performance of ARC-HBR improved substantially (C-statistic 0.72), becoming comparable to PRECISE-DAPT (0.74, *p* = 0.53) [34].

Additional insights were provided by the Osaka Acute Coronary Insufficiency Study (OACIS), a large prospective registry including over 12,000 patients. When stratified by ARC-HBR criteria, the risk of major bleeding during the acute phase exceeded that of recurrent myocardial infarction across all groups, and in ARC-HBR patients, fatal bleeding and fatal myocardial infarction risks were closely balanced. Among hospital survivors, long-term fatal bleeding was rare, whereas recurrent ischemic events remained more frequent. These findings highlight the value of ARC-HBR not only for bleeding risk identification but also for informing early clinical trade-offs in STEMI management [35].

Consistent results were observed in a Chinese multicenter cohort of 1013 ACS patients undergoing PCI with DAPT. ARC-HBR identified a subgroup with markedly higher in-hospital bleeding rates compared with non-HBR patients (15.8% vs. 2.0%, *p* < 0.001). Discriminatory performance of ARC-HBR was superior to that of CRUSADE overall, and particularly strong within the STEMI subgroup, supporting its applicability across diverse populations [34].

In summary, ARC-HBR performs consistently in identifying STEMI patients at increased risk of major bleeding across different healthcare systems. However, its overall discriminative ability remains moderate in this specific population. These observations support the need for further refinement of existing criteria or the development of bleeding risk models specifically tailored to the STEMI setting, particularly in patients with extreme clinical complexity.

### 3.3. PARIS (Patterns of Non-Adherence to Antiplatelet Regimens in Stented Patients) Registry

The PARIS registry was a prospective, multicenter observational study designed to evaluate thrombotic and bleeding risks as well as patterns of dual antiplatelet therapy (DAPT) adherence following percutaneous coronary intervention with drug-eluting stents. A total of 5018 patients were enrolled from 15 centers in the United States and Europe between 2009 and 2010. Importantly, patient enrollment occurred during the index hospitalization after successful PCI with drug-eluting stent implantation. Following enrollment, patients were prospectively followed for up to two years to evaluate ischemic and bleeding outcomes and to characterize patterns of DAPT discontinuation. Using this cohort, investigators developed both the PARIS thrombotic risk score and the PARIS bleeding risk score to stratify patients according to their risks of coronary thrombotic events and major bleeding after PCI. The thrombotic risk score incorporated six clinical variables (diabetes mellitus, acute coronary syndrome at presentation, current smoking, impaired renal function, prior PCI, and prior coronary artery bypass grafting) to estimate the likelihood of myocardial infarction or stent thrombosis during follow-up. By enabling early risk stratification at the time of hospitalization for PCI, the PARIS risk scores were proposed as tools to help clinicians individualize the duration and intensity of antiplatelet therapy based on a patient’s competing risks of thrombosis and bleeding [15].

## 4. In-Hospital Management Strategies for Bleeding Reduction in STEMI Patients

**Figure 1:**
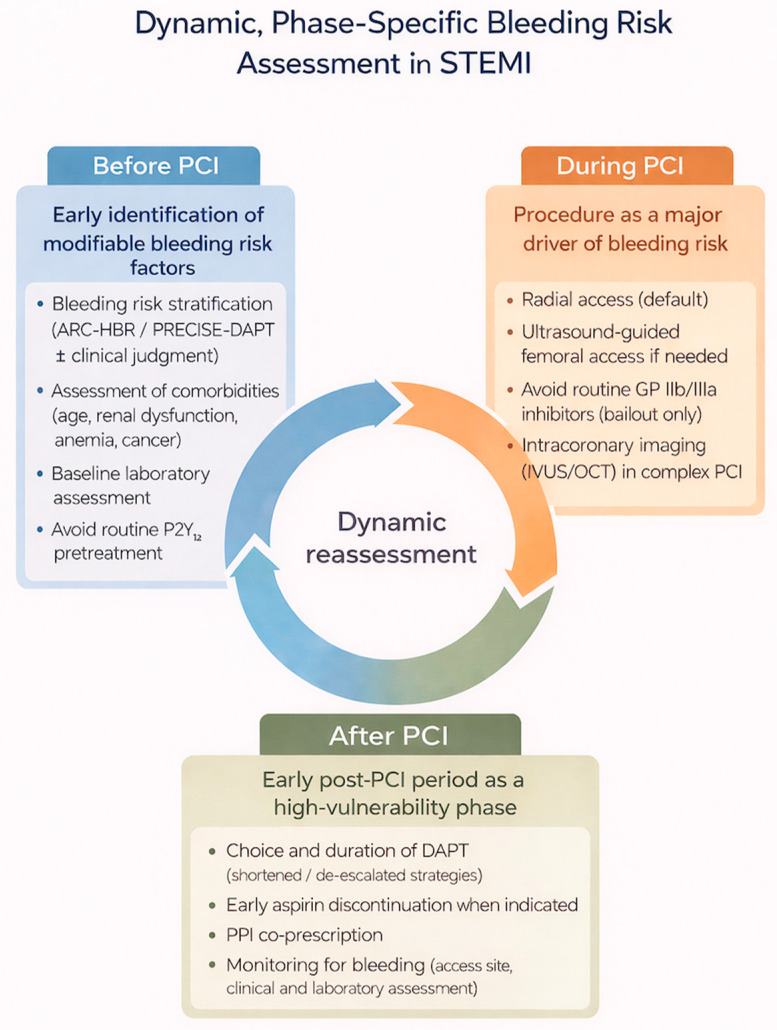
Periprocedural Strategies to Minimize Bleeding Risk in PCI. STEMI: ST-segment elevation myocardial infarction; PCI: percutaneous coronary intervention; ARC-HBR: Academic Research Consortium for High Bleeding Risk; DAPT: dual antiplatelet therapy; PPI: proton pump inhibitor; IVUS: intravascular ultrasound; OCT: optical coherence tomography; GP IIb/IIIa: glycoprotein IIb/IIIa.

In patients presenting with STEMI, clinicians face a dual challenge: preventing thrombotic complications while minimizing bleeding risk. As bleeding has emerged as a prognostically relevant event, contemporary management strategies emphasize a multifactorial and phase-specific approach to care, encompassing the pre-procedural, periprocedural, and post-procedural phases of PCI (Figure 1) [36,37].

### 4.1. Initial Assessment on Presentation

A comprehensive bleeding risk assessment should include a detailed clinical history, encompassing prior bleeding events, current medications (especially anticoagulants and antiplatelet therapies) and relevant comorbidities such as renal or hepatic dysfunction, in addition to the patient’s age and body weight. Baseline laboratory evaluations, including hemoglobin, hematocrit, creatinine, platelet count, and liver function tests, are essential for accurate risk stratification.

### 4.2. Timing of P2Y_12_ Inhibitors

In patients with NSTEMI, substantial evidence has demonstrated a lack of clinical benefit from routine pretreatment with P2Y_12_ inhibitors, with some studies reporting an increased risk of bleeding when these agents are administered before coronary angiography. On this basis, routine pretreatment is not recommended in this setting [38].

In STEMI, available evidence similarly fails to demonstrate a reduction in ischemic events with routine pretreatment. Studies including the ATLANTIC trial and data from the BERN-PCI and SWEDEHEART registries showed no ischemic benefit associated with P2Y_12_ inhibitor pretreatment [39,40,41]. Consequently, current clinical practice guidelines assign a low level of recommendation to this strategy, discouraging its routine use in all patients. Although these studies did not show a significant increase in bleeding, they were not specifically designed to evaluate high bleeding risk populations; therefore, in the absence of ischemic benefit, avoiding routine pretreatment appears particularly appropriate in patients at increased bleeding risk.

When the balance between thrombotic and bleeding risk is uncertain in P2Y_12_-naïve patients, intravenous cangrelor may represent a valuable alternative. In a prespecified pooled patient-level analysis of the CHAMPION-PCI, CHAMPION-PLATFORM, and CHAMPION-PHOENIX trials, including 24,910 patients undergoing PCI for STEMI, NSTE-ACS, or stable coronary disease, cangrelor administered at the time of PCI significantly reduced periprocedural thrombotic complications, including stent thrombosis, compared with clopidogrel or placebo [42,43,44]. Importantly, cangrelor was not associated with an increase in major bleeding or transfusion rates, although a higher incidence of minor bleeding was observed.

Supporting these findings, a single-center propensity score–matched analysis from the CAST registry compared downstream cangrelor administered after coronary angiography with upstream oral ticagrelor pretreatment in P2Y_12_-naïve STEMI patients undergoing primary PCI. No significant differences were observed in in-hospital major adverse cardiovascular events, and downstream cangrelor was not associated with a higher risk of major bleeding, with similar rates of BARC-defined bleeding between strategies [45].

Taken together, these data support the use of intravenous cangrelor as a safe and effective antiplatelet option in selected STEMI patients at high bleeding risk undergoing PCI. This strategy provides rapid and potent platelet inhibition without an excess of major bleeding, allows immediate discontinuation in the event of bleeding, and facilitates subsequent de-escalation to less potent oral P2Y_12_ inhibitors when appropriate.

### 4.3. Choice of P2Y_12_ Inhibitor (Clopidogrel vs. Prasugrel vs. Ticagrelor)

Among oral P2Y_12_ inhibitors used in ACS, clopidogrel, prasugrel, and ticagrelor differ substantially in antiplatelet potency, clinical efficacy, and bleeding risk. Clopidogrel provides less potent and more variable platelet inhibition, partly due to genetic polymorphisms and drug–drug interactions, whereas prasugrel and ticagrelor achieve more rapid, potent, and predictable platelet inhibition and reduce ischemic events compared with clopidogrel in large randomized trials. However, this enhanced antithrombotic efficacy is consistently associated with an increased risk of bleeding [46].

Prasugrel is linked to higher rates of major and life-threatening bleeding, particularly in older patients, those with low body weight, or a prior history of cerebrovascular events, in whom its use is contraindicated or requires dose adjustment [47]. Ticagrelor is also associated with increased non–CABG-related bleeding compared with clopidogrel, although without a significant increase in fatal bleeding, with bleeding risk influenced by age, renal dysfunction, and concomitant anticoagulation [48]. Consequently, selection among P2Y_12_ inhibitors requires careful balancing of ischemic protection against bleeding risk, with clopidogrel often favored in patients at high bleeding risk and prasugrel or ticagrelor preferred in those with high thrombotic risk and acceptable bleeding profiles.

Evidence supporting this tailored approach comes from the POPular AGE trial, which enrolled elderly patients with ACS. In this study, clopidogrel was associated with a significantly lower incidence of bleeding, including major and non–CABG-related bleeding, while achieving non-inferiority for net clinical benefit compared with ticagrelor or prasugrel. These findings support clopidogrel as a reasonable alternative to more potent P2Y_12_ inhibitors in elderly patients, particularly those at increased bleeding risk [49].

In parallel with growing interest in abbreviated and de-escalated antiplatelet strategies, recent studies have explored dose modulation of potent P2Y_12_ inhibitors. In a randomized crossover trial, ticagrelor 60 mg twice daily achieved platelet inhibition comparable to the standard 90 mg dose, with improved tolerability and a more favorable safety profile. This approach may represent a pragmatic strategy to mitigate bleeding risk in selected elderly or high-risk patients without compromising antiplatelet efficacy [50].

Genotype-guided antiplatelet therapy represents another personalized strategy to balance ischemic and bleeding risks. In the POPular Genetics trial, a CYP2C19 genotype–guided approach was noninferior to standard treatment with prasugrel or ticagrelor for thrombotic outcomes and was associated with a significantly lower incidence of bleeding, driven mainly by reductions in minor bleeding. These data support the use of genotype-guided P2Y_12_ inhibitor selection as an effective bleeding avoidance strategy in STEMI patients undergoing primary PCI [51].

### 4.4. Vascular Access

Transradial access has consistently been associated with a lower risk of bleeding compared with transfemoral access, a benefit that is particularly relevant in patients with STEMI. In this setting, where intensive antithrombotic therapy is routinely required, access-site bleeding has a strong impact on clinical outcomes. In the MATRIX trial, which evaluated bleeding avoidance strategies in patients with acute coronary syndromes, transradial access in the STEMI subgroup was associated with significant reductions in major bleeding, all-cause mortality, and net adverse clinical events compared with transfemoral access, reinforcing the central role of vascular access choice in bleeding prevention [52,53].

When femoral access is unavoidable, meticulous procedural technique is essential to minimize vascular complications. Ultrasound-guided femoral puncture improves first-pass success and reduces inadvertent high or low arterial punctures, translating into lower bleeding rates compared with landmark-based approaches [54,55]. The use of micropuncture techniques further limits arterial trauma and reduces the risk of hematoma, pseudoaneurysm, and retroperitoneal hemorrhage [54]. In addition, vascular closure devices facilitate faster hemostasis and early mobilization and have been associated with a reduction in access-site bleeding, particularly in patients receiving potent antithrombotic therapy [56].

### 4.5. Complexity of Procedure

The complexity of PCI procedures has a direct impact on both ischemic and bleeding outcomes in patients with acute coronary syndromes. More complex interventions—such as treatment of multiple lesions, bifurcation stenting, chronic total occlusions, or the use of multiple adjunctive devices—are associated with longer procedural times, higher contrast volumes, and increased rates of vascular complications, all of which contribute to an elevated bleeding risk, particularly in elderly patients and those with impaired renal function [12,15,52].

Whenever clinically feasible, minimizing unnecessary procedural complexity is therefore a key strategy to optimize safety. Simplified procedural approaches, careful planning of lesion treatment, use of smaller sheath sizes, and restriction of arterial punctures reduce vascular trauma, shorten procedure duration, limit contrast exposure, and preserve renal function, translating into improved overall outcomes.

At the same time, when complex PCI is unavoidable, intracoronary imaging should be systematically considered to optimize stent sizing and expansion, reduce malapposition and edge-related complications, and enhance long-term procedural success. This strategy is particularly relevant in patients at high bleeding risk, in whom shorter or de-escalated antiplatelet regimens are frequently pursued. In this context, maximizing procedural “mechanical” safety becomes essential to mitigate ischemic events despite reduced antithrombotic intensity [57,58].

### 4.6. Periprocedural Monitoring and Adjustment

- Adjust post-PCI anticoagulants/dosing in cases of renal impairment, low body weight, or if bleeding signs appear.
- Check hemoglobin, urine/stools (occult), and signs of access-site hematoma.
- Monitor for bleeding in the catheterization lab (ACT) immediately after procedure and over the next 24–48 h.
- Temporarily hold or reduce doses of antithrombotic agents if bleeding risk outweighs benefit.

In patients with STEMI at HBR, meticulous periprocedural monitoring and timely therapeutic adjustments are essential to balance ischemic protection against hemorrhagic complications. Continuous hemodynamic surveillance, including invasive blood pressure monitoring in unstable patients, allows early identification of hypotension or occult bleeding during primary PCI. Serial assessment of hemoglobin and hematocrit levels, although limited by delayed kinetics, may aid in detecting clinically relevant blood loss when interpreted alongside procedural factors and clinical signs. Renal function should be closely monitored, given its influence on antithrombotic drug clearance and bleeding propensity [12,26].

Antithrombotic therapy requires dynamic adjustment throughout the procedure. Weight- and renal function–adjusted dosing of unfractionated heparin is preferred, with activated clotting time (ACT)-guided titration to avoid excessive anticoagulation. Routine use of glycoprotein IIb/IIIa inhibitors should be avoided in high-bleeding-risk patients, reserving their administration for bailout situations. When intravenous P2Y_12_ inhibition is utilized, careful transition to oral agents is warranted to prevent overlapping platelet inhibition [59]. Collectively, an individualized, monitoring-driven approach during the periprocedural phase is critical to optimizing outcomes in high-bleeding-risk STEMI patients.

### 4.7. Red Blood Cell Transfusion Strategies

In patients with acute coronary syndromes, red blood cell transfusion is consistently associated with adverse clinical outcomes and should therefore not be considered a benign intervention [60]. Although the optimal hemoglobin threshold remains debated, available evidence supports a generally restrictive transfusion strategy in most patients. Liberal transfusion at higher hemoglobin levels (9–10 g/dL) has been associated with worse outcomes in observational studies, reinforcing caution against routine early transfusion [61].

Randomized evidence from the MINT trial showed no overall reduction in death or recurrent myocardial infarction with a liberal strategy compared with a restrictive approach. While subgroup analyses did not demonstrate a statistically significant interaction, the absence of clear benefit from liberal transfusion—and the lack of a signal favoring a restrictive strategy in patients with type 1 myocardial infarction—underscores the need for careful clinical judgment rather than protocolized thresholds [62].

Taken together, these data support a restrictive transfusion strategy as the default approach in ACS, with selective deviation in patients with severe anemia, hemodynamic instability, or ongoing ischemia despite optimal medical and revascularization therapy. Transfusion decisions should be individualized, integrating hemoglobin level, ischemic burden, bleeding risk, and overall clinical status, rather than relying on fixed numerical thresholds.

### 4.8. Choice of Anticoagulation During Percutaneous Coronary Intervention

Periprocedural anticoagulation during PCI has a major impact on both bleeding and ischemic outcomes in patients with STEMI. Early randomized trials showed that bivalirudin was associated with lower rates of major bleeding compared with unfractionated heparin, particularly when the comparator strategy included routine use of glycoprotein IIb/IIIa inhibitors; however, this benefit was offset by a higher incidence of acute stent thrombosis [63]. In contemporary practice, where radial access is widely adopted and glycoprotein IIb/IIIa inhibitors are used selectively, randomized evidence has shown no clear net clinical advantage of bivalirudin over unfractionated heparin, with persistent concerns regarding stent thrombosis [52].

Accordingly, unfractionated heparin remains the anticoagulant of choice during primary PCI for STEMI, as endorsed by current ESC guidelines [14]. Its rapid onset of action, reversibility, low cost, and feasibility of ACT-guided titration allow effective anticoagulation with an acceptable bleeding profile when appropriately dosed.

### 4.9. Strategies for Patients Who Need Concurrent Anticoagulation

Patients requiring chronic oral anticoagulation who present with acute coronary syndromes and undergo PCI represent a particularly challenging population, as they combine a high thrombotic risk with a substantial risk of bleeding. This scenario is most frequently encountered in patients with atrial fibrillation, mechanical heart valves, or prior venous thromboembolism, and poses additional complexity in STEMI, where urgent reperfusion and potent antithrombotic therapy are required.

In patients receiving chronic oral anticoagulation, contemporary evidence supports performing PCI without interruption of vitamin K antagonists or non–vitamin K oral anticoagulants, avoiding bridging strategies that increase bleeding without improving ischemic outcomes. In patients treated with vitamin K antagonists, additional unfractionated heparin should generally be avoided when the INR is >2.5, whereas reduced-dose heparin may be considered when the INR is between 2.0 and 2.5. In contrast, in patients receiving non–vitamin K oral anticoagulants, adjunctive low-dose parenteral anticoagulation during PCI (e.g., unfractionated heparin 50–70 IU/kg) is recommended regardless of the timing of the last dose, as oral anticoagulation alone does not provide reliable intraprocedural anticoagulation [64]. Although evidence in STEMI is derived mainly from observational data and sub-analyses of AF-PCI trials, these principles are generally applicable in this setting [65].

Given the heightened bleeding risk, antiplatelet strategies in patients requiring concurrent anticoagulation should prioritize bleeding avoidance. Clopidogrel is generally preferred over more potent P2Y_12_ inhibitors, particularly in patients at high bleeding risk.

In the post-procedural phase, randomized trials in AF-PCI populations—including RE-DUAL PCI, AUGUSTUS, and ENTRUST-AF PCI—have consistently demonstrated that dual therapy with a non–vitamin K oral anticoagulant plus a P2Y_12_ inhibitor significantly reduces bleeding compared with triple therapy, without an excess of ischemic events. Consequently, contemporary practice has shifted toward very short courses of triple therapy, limited to the acute periprocedural period (often up to one week), followed by early transition to dual therapy in most patients.

Risk stratification tools may further aid treatment individualization in this complex population. In patients with atrial fibrillation undergoing PCI, application of the PRECISE-DAPT score within the RE-DUAL PCI framework has been shown to identify individuals at increased bleeding risk and to inform the intensity and duration of antithrombotic therapy [66]. This approach facilitates a more precise balance between bleeding and ischemic risk, supporting individualized decision-making and optimization of the benefit–risk profile.

### 4.10. Adjunctive Measures and Bleeding Prophylaxis

Gastrointestinal protection with proton pump inhibitors, especially when aspirin is used, is strongly advised. In a randomized, double-blind trial involving patients with coronary artery disease receiving dual antiplatelet therapy with clopidogrel and aspirin, the concomitant use of omeprazole was evaluated for its effects on cardiovascular and gastrointestinal outcomes. The addition of omeprazole did not result in a significant increase in major adverse cardiovascular events, including cardiovascular death, myocardial infarction, or stroke, compared with placebo. In contrast, omeprazole therapy was associated with a significant reduction in gastrointestinal events, particularly upper gastrointestinal bleeding. These results indicate that omeprazole provides effective gastrointestinal protection without compromising cardiovascular safety in patients treated with clopidogrel [67].

Novel agents like subcutaneous P2Y_12_ inhibitors (e.g., selatogrel) and reversal agents for ticagrelor are under investigation and may enhance safety in the future.

## 5. Conclusions and Future Directions

Bleeding risk in STEMI is a major determinant of prognosis and should be addressed as an integral component of contemporary management rather than a secondary safety consideration. Although validated bleeding risk scores provide a useful starting point, they do not fully capture the dynamic nature of bleeding risk during hospitalization, which is strongly influenced by procedural complexity, antithrombotic strategies, and evolving clinical status. Effective care therefore requires a phase-specific and continuously adaptive approach, integrating structured risk assessment with meticulous procedural planning and close clinical monitoring.

Future directions should focus on refining existing bleeding risk models and developing dynamic, context-specific tools capable of integrating clinical, procedural, and laboratory variables in real time. Advances in digital health, intracoronary imaging, and artificial intelligence may facilitate more precise risk prediction and support personalized decision-making throughout the hospital course. Ultimately, embedding dynamic bleeding risk reassessment into routine STEMI care pathways represents a critical step toward safer, more individualized, and more effective management of this high-risk population.

## Figures and Tables

**Table 1:** Current Risk scoring systems for in-hospital bleeding generated and validated in STEMI.

Score	STEMI Applicability	No. of Patients	Study Enrollment Period	Key Variables	Predictive Performance (C-Statistic/C-Index)	Timing of Assessment	Bleeding Endpoint	Clinical Utility in STEMI	Key Limitations
CRUSADE	Limited (STEMI under-represented)	n ≒ 89,000	2003–2006	Hematocrit, creatinine, heart rate, sex, comorbidities	0.77	At presentation	In-hospital major bleeding	Early risk estimation before angiography	Primarily developed in NSTEMI; limited relevance in contemporary primary PCI
ACTION	Yes	n ≒ 90,000 (STEMI & NSTEMI)	2012–2013	Age, creatinine clearance, heart rate, weight, blood pressure, comorbidities	0.78	At presentation	In-hospital major bleeding	Admission-based stratification applicable to STEMI	Relatively complex; modest incremental value over clinical judgment
ACUITY-HORIZONS	Yes (HORIZONS-AMI)	n ≒ 13,800 (ACUITY) n ≒ 3600 (HORIZONS-AMI)	ACUITY: 2003–2005 HORIZONS-AMI: 2005–2007	Anticoagulation regimen, age, sex, creatinine, baseline hemoglobin	0.70	Periprocedural	Non-CABG-related major bleeding	Integrates procedural and pharmacologic bleeding risk during primary PCI	Trial-derived; limited generalizability to real-world STEMI populations
SWEDEHEART	Yes	n = 97,597	2009–2014	Hemoglobin, age, sex, renal function, C-reactive protein	0.80	Early hospitalization	In-hospital major bleeding	Contemporary, real-world STEMI risk stratification	Limited external validation; regional derivation

## Data Availability

No new data were created or analyzed in this study. Data sharing is not applicable to this article.

## Abbreviations

The following abbreviations are used in this manuscript:

STEMI	ST-segment elevation myocardial infarction
ACS	Acute coronary syndrome
BARC	Bleeding Academic Research Consortium
PCI	Percutaneous coronary intervention
DAPT	Dual antiplatelet therapy
ACT	Activated clotting time
HBR	High bleeding risk
CABG	Coronary artery bypass graft
